# Parental involvement intervention: effect on students’ self-efficacy and math achievement among a Nigerian sample

**DOI:** 10.3389/fpsyg.2025.1589069

**Published:** 2025-07-22

**Authors:** Olutola Opeyemi Akindipe

**Affiliations:** Department of Psychological and Brain Sciences, University of North Florida, Jacksonville, FL, United States

**Keywords:** parental involvement, self-efficacy, math, achievement, intervention

## Abstract

Although math underachievement remains a global educational challenge, empirical evidence indicates that parental involvement interventions have significantly improved students’ performance in the subject. The problem, however, seems to persist in Nigeria where there are reports of low parental involvement and no extant studies of parental involvement intervention in math. This study examined the impact of parental involvement intervention on students’ math achievement and self-efficacy. The sample consisted of 51 fifth grade students recruited from 2 private schools in the Lagos educational district III that chose to participate in the study. Comprising 17 boys and 34 girls with an average age of 10.89 years, students from one of the schools served as the intervention group while the second school was the control group. The intervention group underwent home structure, parental supervision, and school-home communication intervention while the control group did not. Collected data was analyzed using analysis of covariance (ANCOVA) and independent t-test analyses. Results showed that the intervention significantly improved students’ math achievement but not math self-efficacy. Educational implications of the results are discussed.

## 1 Introduction

Parental involvement refers to the different activities parents engage in to help their children succeed. Because of its integral role in the education of students, it continues to receive attention from scholars and researchers alike. Although there is no universal agreement to what parental involvement specifically entails, Epstein (1995) identified six major types of parental involvement activities. These are parenting, communicating, volunteering, learning at home, decision making, and collaborating with the community. Similarly, Pomerantz et al. (2007) classified parental involvement into school or home-based parental involvement. Home-based involvement includes homework assistance, parental support, parental expectations, and extracurricular activities while school-based involvement includes parental volunteering and Parents Teachers Association attendance among others.

Although there are a few conflicting findings associated with parental involvement, its importance to students’ educational success has been generally outlined by studies (Ho, 2010; Pomerantz et al., 2007; Grolnick and Slowiaczek, 1994) and spans beyond elementary and middle school to higher education (Izzo et al., 1999; Epstein, 1995). Parental involvement influences several attributes of learning such as interest, attitude, motivation, self-regulation, and self-efficacy beliefs (Khajehpour and Ghazvini, 2011; Jeynes, 2005; Zimmerman, 2000; Ho, 2010; Friedel et al., 2010). Involved parents guide, support, and encourage their children’s academic effort and pursuits. They communicate and model appropriate educational norms, aspirations, and values which increase students’ academic confidence and motivation. Also, parental involvement enhances students’ strategy use, academic resilience, and performance (Pomerantz et al., 2007; Ponce-Garcia and Madewell, 2024; Kovács et al., 2022). Its relevance cuts across all subjects, including math, where it influences students’ expectation of math success, choices, and supports the development of positive math identity (Sheldon and Epstein, 2005; Fan and Williams, 2010; Turner et al., 2004).

Nevertheless, students’ math achievement remains a global challenge, affecting students from all cultures and backgrounds (Foley et al., 2017). Many elementary, middle, and high school students in the United States and around the world continue to struggle in math (Stigler et al., 2010), resulting in low proficiency and underachievement in the subject. Consequently, this has led to reduced students’ admission, enrollment, and graduation rates from Science, Technology, Engineering, and Mathematics (STEM) disciplines in higher education (Perna and Titus, 2005; Salman, 2001; Nicholas et al., 2015). The negative impact is greater for countries in the Global South, such as Nigeria, which has one of the largest economies (GDP) and the largest population in Africa (You et al., 2020). Despite its potential for greater technological innovation, and advancement, Nigeria’s educational system is greatly challenged (Adeniran et al., 2020). Students’ performance in STEM courses, especially math, at all educational levels in Nigeria is poor (Nzeadibe et al., 2019, 2020). For example, less than 40 % of students in the country who sat for the West Africa Certificate math examination from 2010 through 2015 had credit or distinction (Onyeka and Arokoyu, 2018).

The implementation of parental involvement interventions in math education has increased in recent times due to research findings reporting that parental involvement improves students’ math proficiency and achievement (Friedel et al., 2010; Sirvani, 2007; Berkowitz et al., 2015; Ketterlin-Geller et al., 2008). Some of these interventions are general interventions, involving education, and training or math specific interventions, focusing on education and training in math (Nye et al., 2006). General interventions tend to educate parents on the importance of parental involvement to increase their engagement while math specific interventions train parents on skills or strategies relating to math activities such as concepts, apps, homework completion and school-home communication among others (Nye et al., 2006; Sirvani, 2007; Jay et al., 2017; Shivraj et al., 2018).

While math specific interventions are more common in Western countries probably due to stronger collaboration between schools and families, such interventions may be inappropriate in Nigeria where parents are often less involved in their children’s education due to daily struggle for economic livelihood and survival (Omoteso, 2010; Kutelu and Olowe, 2013). Interventions that incorporate both general and math specific intervention may be more relevant in this less industrialized nation given the need to increase parents’ awareness of parental involvement and to teach them specific math skills they can utilize with their children.

Understanding students’ sociocultural environment is fundamental to the success of any parental involvement intervention. Cultural and socioeconomic differences based on geographical location affects parental involvement and intervention in students’ math achievement (Wang and Wei, 2024; Wang et al., 2024; Xu et al., 2024). In the traditional Nigerian culture, the family extends beyond the nuclear unit to include members of the extended family such as grandparents, uncles, and aunts, who often live together. These relatives are a significant part of students’ lives because they are involved in their care and upbringing. As a collectivistic culture, Nigerians do not only value the collective self, conformity to group’s norms, communal living, and relationship, but also hierarchical order (Hofstede, 2001; Gorodnichenko and Roland, 2012; Triandis, 1995). Although the family is the primary in-group and extremely valued, the community is also seen as an extension of the family. While individuals hold the strongest allegiance to the family, they also respect and honor other members of the community, especially elders and leaders.

Although the average Nigerian parent values education and views it as a leverage for success and fulfillment in life, however, many of them are rarely involved in their children’s education (Ayeni, 2021; Angwaomaodoko, 2023). Parents who are fully employed or own businesses have little or no time for their children’s education, however, those more financially buoyant often enroll their children in afterschool learning or home tutoring. Uneducated parents are less involved in their children’s education because they lack the requisite knowledge necessary for participation. Such parents tend to avoid their children’s school more than other parents. Because school officials and teachers are revered as authority figures within the community (Allik and Realo, 1996), parents are often reluctant to interact with them because they do not want to be perceived as intrusive or questioning their authority or competence (Araujo, 2009; Colombo, 2006). Therefore, they rarely visit their children’s schools except at the school’s invitation, which are usually for special events, the Parents Teachers Association (PTA) meetings, or students’ behavioral issues (Gonzalez-DeHass et al., 2005).

Although several research have examined the influence of parental involvement on the math achievement of Nigerian students, all of them to the best of my knowledge, have been descriptive or correlational. There are no existing parental involvement intervention studies on math achievement. For example, Olatoye and Agbatogun (2009) investigated the relationship between parental involvement and students’ math achievement. Also, Adeyeye (2024) examined the influence of parental involvement on the math achievement of high school students. Similarly, Akindipe (2015) in a study examining the influence of parental involvement on math achievement motivation found positive association between parental involvement and math achievement motivation, parental involvement and math students’ self-efficacy, math self-efficacy and math achievement motivation, and cultural orientation and math achievement motivation among Nigerian elementary school students. The study reported that parents’ involvement was low across all dimensions of parental involvement, particularly school-home communication, home structure, and parental supervision. Several studies have corroborated the low involvement of Nigerian parents across the dimensions of parental involvement identified by Akindipe (2015). Angwaomaodoko (2023) reported that Nigerian parents rarely monitored their children’s work and volunteered in school. Also, he stated that only rich parents created an enabling home environment for their children’s learning, yet they seldom monitor the work themselves. Relatedly, other studies concluded that home structure is positively related to students’ math achievement (e.g., Ayeni, 2021) and recommended for the communication of students’ academic records to parents (Esho et al., 2025).

Contributing to students’ math achievement is self-efficacy. Postulated by Bandura (1986), self-efficacy (SE) is an individual’s belief or perceived capability to perform a given or specific task at a desired level (Schunk, 1991). It is considered the most significant predictor of students’ achievement (Schunk and Miller, 2002; Britner and Pajares, 2006) probably due to its crucial role in influencing students’ affective and cognitive states during learning (Bandura, 2012; Britner and Pajares, 2006; Phan, 2009). Also, it is a good predictor of students’ reflective thinking, self-regulatory strategies, and problem-solving abilities (Phan, 2009; Zimmerman, 2000). In addition, it has been positively associated with math achievement (Britner and Pajares, 2006; Ramdas and Zimmerman, 2000). Students with higher math self-efficacy spend more time and effort on math practice and persevere on difficult problems (Margolis and McCabe, 2004).

Bandura (1986) postulated that individuals develop self-efficacy from four primacy sources namely mastery experiences, vicarious experiences, verbal persuasion, and physiological arousal. Mastery experience relates to individuals’ successes at performing specific tasks. Bandura (1997) opined that mastery experience is the most influential source of self-efficacy because it provides the best evidence that an individual can successfully perform a task. Vicarious experiences occur when individuals observe the resultant rewards that follow others’ successful execution of tasks. Verbal persuasion includes responses, feedback, or evaluation received from others such as parents, teachers, and friends, during or after task performances which increase self-efficacy. Physiological arousal is the bodily sensation individuals experience before or during task performances which influences the successful performance of tasks. Information from these sources is cognitively interpreted to influence self-efficacy. Among collectivistic cultures, though, verbal persuasion and vicarious experiences are the most profound sources of self-efficacy development (Klassen, 2004; Ahn et al., 2016).

From the foregoing, parental involvement inevitably influences the development of students’ self-efficacy. Parents’ attendance and participation in school programs, involvement in extracurricular activities, and assistance with homework among others provide great opportunities for students to gain mastery and vicarious experiences and to receive verbal persuasion. Also, parents’ repeated encouragement, support, and valuable feedback to students during academic and extracurricular pursuits unconsciously communicates the importance of education which increases self-efficacy (Friedel et al., 2010; Fan and Williams, 2010; Kung and Lee, 2016; Williams et al., 2017).

The empirical findings of some Western studies on parental involvement interventions have been conflicting. For example, some studies reported that the intervention improved students’ math achievement (e.g., Destin and Svoboda, 2017; Kiger et al., 2012), other studies reported inconclusive evidence (Desforges and Abouchaar, 2003; Fishel and Ramirez, 2005), while a few others concluded it had negative effect on math achievement (Levpušček and Zupančič, 2009; Silinskas and Kikas, 2019). These inconsistent findings have raised concerns and necessitated more investigation into the effectiveness of interventions.

This current study investigates the effect of parental involvement intervention on students’ math self-efficacy and achievement among a Nigerian sample. It was conducted as an extension of Akindipe’s (2015) study, and its primary purpose was twofold. First, to implement intervention for parents in the dimensions they had the lowest involvement, namely home structure, parental supervision, and school-home communication. Second, to examine the effect of the intervention on students’ math achievement and self-efficacy. Incorporating both general and math specific intervention, the study included an awareness training for parents on the importance of parental involvement and specific training for math parental supervision, home structure, and school-home communication. Home structure was operationalized as students having well-illuminated space with reading table and chair for math practice at home; parental supervision was defined as parents’ active monitoring of students’ math work for at least 15 min each day, and school-home communication as parents receiving, signing, and returning weekly reports of students’ math progress to the school. Math achievement and math self-efficacy were operationalized as students’ scores on the math posttest and self-efficacy measures respectively, with higher score representing higher achievement and self-efficacy and lower score indicating the opposite.

To achieve its aim, the study addressed the following research hypotheses:

*H1*: Parental involvement intervention will have a significant effect on students’ math achievement.

*H2*: Parental involvement intervention will have a significant effect on students’ math self-efficacy.

## 2 Methods

### 2.1 Research design

The intervention utilized a quasi-experimental research design. The participating schools were two private elementary schools selected from the Lagos educational district III based on the headteachers’ willingness to participate in the study. One of the schools served as the intervention school while the other was the control group.

### 2.2 Participants

The participants were 51 fifth grade students who self-selected themselves to participate in the study. Their eligibility was based/premised on scoring less than 15 points on a self-report survey that assessed their parent’s involvement in assisting them complete their math assignment at home and signing the consent form. The survey asked questions about the availability of a study place at home, parents’ supervision and scheduling of their math assignments at home and the extent of school-home communication about math learning in school. An example of an item was “Does your child have a regular study place or area for mathematics practice at home? If yes, how often is this place or area used daily?” The maximum obtainable point was 30 and students that scored above 15 were classified as having high parental involvement in math and therefore excluded from the study. The participants also completed the assent form. Their age ranged from 9 to 14 years with an average of 10.89 years. 17 of the students were male (33.4%) while 34 were female (66.6%). The parent-participants age was between 31 and 55 years. 10% (3) of them were between 31 and 35 years, 12 (80%) were between 36 and 45 years, 2 (6.66%) were between 46 and 50 years, and 1 (3.33%) was between 51 and 55 years. 40% of them refused to indicate their age. 52. 9% of them were mothers, 18 of them were fathers (35.3%) while 6 did not state their gender (11%). Four (7.8%) of the parents did not give their educational qualification, 3 (5.9%) had a vocational or polytechnic education, while 44 (86.3%) had a minimum of a bachelor’s degree. Although the study originally started with 56 participants, only 51 of them completed the study due to attrition. The intervention group comprised 21 participants while the control group had 30 participants.

### 2.3 Procedure

After obtaining the Institutional Review Board (IRB) and state approvals from the appropriate authorities. I contacted 8 schools within the Lagos educational district III but only 2 headteachers were willing to participate in the study. This high rate of refusal among school administrators in supporting interventions because of disruptions to teaching schedules, clashes with instructional time, and stress given many schools’ low resources and understaffing issues has been cited in literature (Nagrale and Jiandani, 2024). The headteachers of the two schools introduced me to the 5^th^ grade teachers and instructed them to assist me. I met with the teachers, two from the first school and three from the second, explained the purpose of the study to them, and asked for assistance in developing and administering weekly math tests to students. The teachers in each school generated 30 math questions with the grading rubric using the state math curriculum. Each math item was matched with its corresponding curricular topic. The two sets of tests were exchanged between the teachers in the two schools, and they were asked to identify items that failed to align with the curriculum. This established construct and content validity. The math tests were used for the pretest, posttest, and the weekly tests.

Parents in the intervention group attended a training session comprising 2 parts. The first part was an awareness training focused on the importance of parental involvement developed using the Involvement Schools Parents and You (I-S-P-Y) parental involvement training manual. The second part involved activities on setting up study space and creating study schedule for students to use at home, completing the school-home communication math progress report, and monitoring students’ math practice all modeled after Canter and Hausner’s (1988) “Homework without Tears” handbook. The training lasted for about 55 min and parents were informed to set up the study space and schedule immediately after they arrived home. Copies of the training handout were given to parents in attendance and sent to parents who missed the training. Parents in the control group did not receive any of the materials. The math pretest was administered to students in both the control and intervention group. Parents in the intervention group were given a week to complete the home set up after which they were notified to begin the intervention.

The intervention group utilized the home structure, underwent parents’ supervision, and has communication between parents and the school for 6 weeks. Specifically, parents ensured that students had a quiet, well-lit study space within the home devoid of distraction and noise for math practice. Also, they received reports of the students’ math test, signed and returned them to school. In addition, they created and monitored students’ use of math study schedule for at least 15 min daily. Also, they posted the study schedule in conspicuous parts of the house such as their rooms, kitchen walls, or fridges, as reminders. They simply monitored students and did not assist them with math practice. Participants in both groups took the weekly math tests, however, only parents in the intervention group received the progress report, signed, and returned them to school. Also, parents in the intervention group received weekly reminders to implement the intervention. The math posttest and self-efficacy measures were administered to students in both groups at the end of the 6 weeks intervention.

### 2.4 Measures

#### 2.4.1 Parental involvement scale

Parental involvement was measured using a multidimensional scale adapted from Fan (2001), Epstein (1995), and Yan and Lin (2005) studies. The scale had 25 items formatted after the 5-point Likert scale, ranging from 5 (strongly agreed) to 1 (strongly disagreed) (e.g., “My parents/guardians always attend school activities that I am involved in”). The scale comprised parental involvement dimensions such as participation in school activities, extracurricular learning, home structure and supervision, school-home communication, and educational aspiration.

#### 2.4.2 Math self-efficacy

The math self-efficacy scale was adapted from Pintrich and De Groot’s (1990) self-efficacy subscale of the Motivated Strategies for Learning Questionnaire (MSLQ). Comprising 9 items on a 5-point Likert scale ranging from 1 (strongly disagreed) to 5 (strongly agreed), it had a high Cronbach alpha value of 0.93 and a predictive validity of 0.41. Items were rephrased to reflect math self-efficacy, e.g., I expect to do very well in this class” was changed to “I expect to do very well in my math class.” Higher scores indicated higher math self-efficacy, and lower scores represented lower self-efficacy.

#### 2.4.3 Math achievement measure

Participants’ math achievement was assessed with 20-item math test developed by the math teachers in the 2 schools. Each test item was matched against the state’s math curricular content to establish content and construct validity (Messick, 1995). The teachers were in complete agreement on the validity of the math test thereby producing evidence of good inter-rater reliability.

### 2.5 Intervention integrity

Six participants comprising 4 female and 2 male students were randomly selected and asked about their use of the study schedule and parents’ math supervision at home. This was to gage parents’ adherence to the implementation of the intervention which could not be directly verified. Their responses showed some variability in parents’ implementation of the intervention. Four participants reported that their parents, at some point, delegated supervision to their older siblings or relatives. Also, four participants said their parents forgot on a few occasions to supervise their practice and that they had to remind them to do so. One participant said that he relocated his study space to the veranda to avoid the noise coming from inside the house. Summarily, participants’ responses reveal that parents and participants improvised in the implementation and sustenance of the intervention to some extent.

## 3 Results

To test the hypothesis that parental involvement intervention influenced students’ math achievement, the intervention and control group were first compared at baseline. Levene’s test of normality and homogeneity of regression revealed that students in the intervention and control groups did not differ on the math pretest (*F* (1, 49) = 0.378, *p* = 0.541). An analysis of covariance (ANCOVA) was conducted to examine the effect of parental involvement intervention on math achievement while controlling for the math pretest (*F* (1, 48) = 9.855, *p* = 0.003, *η*_p_^2^ = 0.17). The findings demonstrated a statistically significant difference in the math achievement of the intervention (*M* = 61.19, SD = 22.07) in comparison to the control group (*M* = 53.67, SD = 22.66). This result shows that the intervention was effective in improving students’ math achievement. The partial eta square was 0.17 indicative of small effect size.

The second hypothesis that the intervention would result in higher math self-efficacy was not supported by the findings. Although Levene’s test showed that the intervention and control groups were equal at baseline as the homogeneity of variances was not significant (*F* (1, 49) = 0.895, *p* = 0.349, ns), an independent t-test analysis that examined the effect of the intervention on math self-efficacy showed no significant difference between the two groups. While the self-efficacy of the intervention group (*M* = 39.29; SD = 3.30) was slightly higher than the control group (*M* = 38.5; SD = 3.09), the difference, however, was not statistically significant (*t* (49) = 0.868, *p* = 0.39, ns). Thus, the intervention did not significantly increase students’ self-efficacy. Cohen’s *d*, effect size, was 0.25 suggesting a small effect size.

## 4 Discussion

The scarcity of parental involvement intervention research in Nigeria and the conflicting result of previous studies on the effectiveness of interventions makes the current study a unique one. Specifically, the study examined the effect of a parental involvement intervention comprising home structure, parental supervision, and home-school communication on students’ math achievement and self-efficacy using a Nigerian student sample.

As hypothesized, the findings demonstrated that the parental involvement intervention was effective in improving the students’ math achievement. Enhancing parents’ general awareness of parental involvement and training them to provide home structure for students’ math practice, supervision, and for school-home collaboration improved students’ math achievement. This result is consistent with previous studies showing that home structure, parental supervision, and school-home communication intervention had a significant effect on students’ math grade, proficiency, or performance (Toney et al., 2003; Kiger et al., 2012; Berkowitz et al., 2015). Although the effect size was small, the low-stake characteristics of the math test, the absence of prior parental involvement interventions involving Nigerian students, and the replicability of the study makes it significant for the population under study.

Contrary to expectations, the result did not show that the parental involvement intervention was effective in increasing students’ math self-efficacy. Although there was a small difference between the math self-efficacy of the intervention and control group, surprisingly, it was not significant. There are a few plausible reasons for this finding. First, previous studies state that students from collectivistic cultures tend to report lower self-efficacy in comparison to their counterparts from individualistic cultures even when they perform better (Yan and Gaier, 1994; Lee, 2009). They attempt to downplay their achievement and avoid self-promotion to foster collective harmony. Therefore, it is possible that participants in the intervention group were being modest in reporting their self-efficacy as culturally expected, thus making their self-efficacy almost at the same level as the control group. Second, although the intervention provided more opportunities for parents to engage with their children, it did not require parents to give feedback to students on their math practice. It is most likely, therefore, that since parents revere school officials as authority figures, they simply obeyed the intervention instruction and provided no form of feedback to the students. Consequently, because the self-efficacy of students from collectivistic culture emanates primarily from verbal persuasion and vicarious experiences of their group members such as parents and relatives than other self-efficacy sources (Klassen, 2004; Ahn et al., 2016), it is possible that parents’ non-provision of verbal persuasion to students prevented the intervention from impacting their self-efficacy and making it significantly different from the control group. An Intervention that incorporates a feedback component or references the vicarious experiences of members of the collective group might possibly have enhanced and reflected in students’ report of self-efficacy significantly above the control group.

## 5 Conclusion

### 5.1 Implications for practice and policy

The findings of this study have several educational implications for parents, school administrators, educators, parents, and policy makers. It demonstrates the need for culturally relevant interventions for parental involvement. For example, future research in collectivistic cultures in Nigeria and the Global South should extend parent involvement beyond parents to other relatives, especially those living in the same house as the student. Given that these members of the extended family are actively involved in raising the children (Ogunola, 2018), this can promote greater parental involvement. Also, the findings reveal that students played an instrumental role in the implementation of the intervention; therefore, they should be actively involved in parental involvement interventions. While this might seem contrary to the cultural expectation within some collectivist cultures, it demonstrates the potency of students’ agency in educational matters and advocates for their active engagement in parental involvement interventions. Finally, it encourages parents to create appropriate home structures and practices that they can easily utilize at home with their children.

### 5.2 Limitations and future research

The following limitations are important to note when interpreting the findings of this study. First, the study was quasi experimental. Although both groups were comparable at baseline, future research may want to conduct an experimental study with participants randomly assigned into the intervention and control group. Second, the sample size was very small due to the refusal of several schools and parents to participate in the study. This limits the external validity of the study, hence its generalizability of the result beyond the sample. Third, it was somewhat impossible to fully ascertain the intervention integrity, especially the quality of parents’ adherence to the intervention. Although 6 participants were interviewed to get a glimpse of intervention adherence, their responses could not be directly verified because it was self-reported. Fourth, the intervention was only implemented for 6 weeks given the time constraints associated with the schools’ academic calendar, especially the need for instructional time and end-of-semester examinations and activities. Although some studies suggest that 4 weeks is sufficient to detect intervention effectiveness (e.g., Bailey, 2006), future studies might want to implement interventions that last longer, probably an academic year. Fifth, students’ self-efficacy was only assessed once, at the end of the intervention. Although Levene’s test of homogeneity of variances showed that math self-efficacy of both groups was comparable at baseline, future studies may want to take a pre-and post-assessment of the construct.

## Data Availability

The raw data supporting the conclusions of this article will be made available by the authors, without undue reservation.
